# Aneurysmal Bone Cyst located in the Mandibular Condyle

**DOI:** 10.1186/1746-160X-5-8

**Published:** 2009-02-16

**Authors:** Sandro Pelo, Giulio Gasparini, Roberto Boniello, Alessandro Moro, Pier Francesco Amoroso

**Affiliations:** 1 Department of Oral and Maxillofacial Surgery, Catholic University of S. Cuore, A. Gemelli Rome. Via G. Moscati 31/33 00168 Rome Italy

## Abstract

A rare case of aneurysmal bone cyst (ABC) located in the mandibular condyle in a 10-year-old boy is presented. The patient came to our attention for a sudden swelling in the right temporomandibular region, the mouth opening was not reduced.

A rapid growing mass, depicting soft tissue invasion, in the right condyle of the mandible was found. Clinically and radiographically it resembled to a malignant lesion. The surgical excision of the mandibular condyle allowed a complete removal of the lesion. The histological examination revealed a pseudocystic expanding osteolytic lesion containing blood-filled space separated by connective tissue and many osteoclastic giant cells, which was a conventional vascular ABC.

The ABC is an infrequent bone lesion which can only be found very rarely at the craniofacial skeleton. There have been described about 160 cases of ABC originated in the molar region or in upper maxilla and even more rare is the location of this cyst in the mandibular condyle. Only 6 cases were reported in the literature to date.

A complete surgical resection of this osteolytic lesion is the treatment of choice considering its high recurrence rate. The condyle was not replaced with any graft. Therefore a functional device was used after surgery to overcome the lack of the condyle and to stimulate the growth of the ramus.

## Introduction

The aneurysmal bone cysts are benign neoformations which can affect all the skeleton bones. More than half occur in the metaphysic of long bones (especially femur and tibia) and between 12 and 30% in the spine. ABC occurs very rarely in the jaws, about 160 cases have been reported and two thirds were located in mandible (the body of the mandible 40%, the ramus 30% and the angle 19%) and one third in the maxilla [[1-3] and [4]]. They represent about 1.5% of all nonodontogenic and nonepithelial cystic of the jaws. Considering all types of jaw cysts the ABC is extremely rare with 0.5%. The average age of occurrence is 13 years and 80% of patients are less than 20 years old with no gender predilection [1,5]. An aneurysmal bone cyst of the condyle is even more unusual, with only 6 cases reported in the literature to date [[6-10], and [11]].

The aneurysmal bone cyst of the jaw is a pseudocystic lesion. It is a rapidly growing and destructive bone lesion characterized by replacement of the normal bone with fibro-osseous tissue containing blood-filled sinusoidal or cavernous spaces. However an unusual solid, focally hemorrhagic variant has recently been reported [2]. The solid type (5% of the cases) may present as a small, asymptomatic lesion first noticed as a radiolucency on a routine radiograph, or a small swelling. Bleeding is not as brisk during surgery with this type of ABC as with the vascular type. The other extreme is the vascular type of ABC (95% of the cases), which manifests as a rapidly growing, expansive, destructive lesion causing cortical perforation and soft tissue invasion. A third form, or "mixed" variant, demonstrates features of both the vascular and solid types. In fact, this may be a transitory phase of the lesion because sudden "activation" or rapid enlargement of stable lesions has been reported [[6,9] and [10]].

The etiology of ABC is unclear and controversial. One theory states that trauma causes an inciting injury to periosteal vessels, thus initiating the development of ABC [12]. However Tillman et al, reporting 95 cases, demonstrated no significant history of antecedent trauma [4]. Jaffé and Lichtenstein refer to alteration in local haemodynamics causing increased venous pressures and engorgement of the vascular bed in the transformed bone, leading to resorption, connective tissue replacement, and osteoid formation [13]. Additional theories about the etiology of this lesion are a subperiosteal intraosseous hematoma [12] or other publications state that the ABC is a secondary phenomena occurring in primary cystic lesions of bone or tumours [14].

Histologically the ABC is considered a pseudocyst due to the absence of an epithelial wall. The ABC is an expanding osteolytic lesion containing blood-filled spaces of variable size, separated by connective tissue by bone trabeculae or osteoid tissue and many osteoclastic giant cells. The lesion does not have any clinical or radiological specificity. Recurrence has been reported confirming the aggressive behaviour of this lesion [3].

## Case report

**Figure 1:**
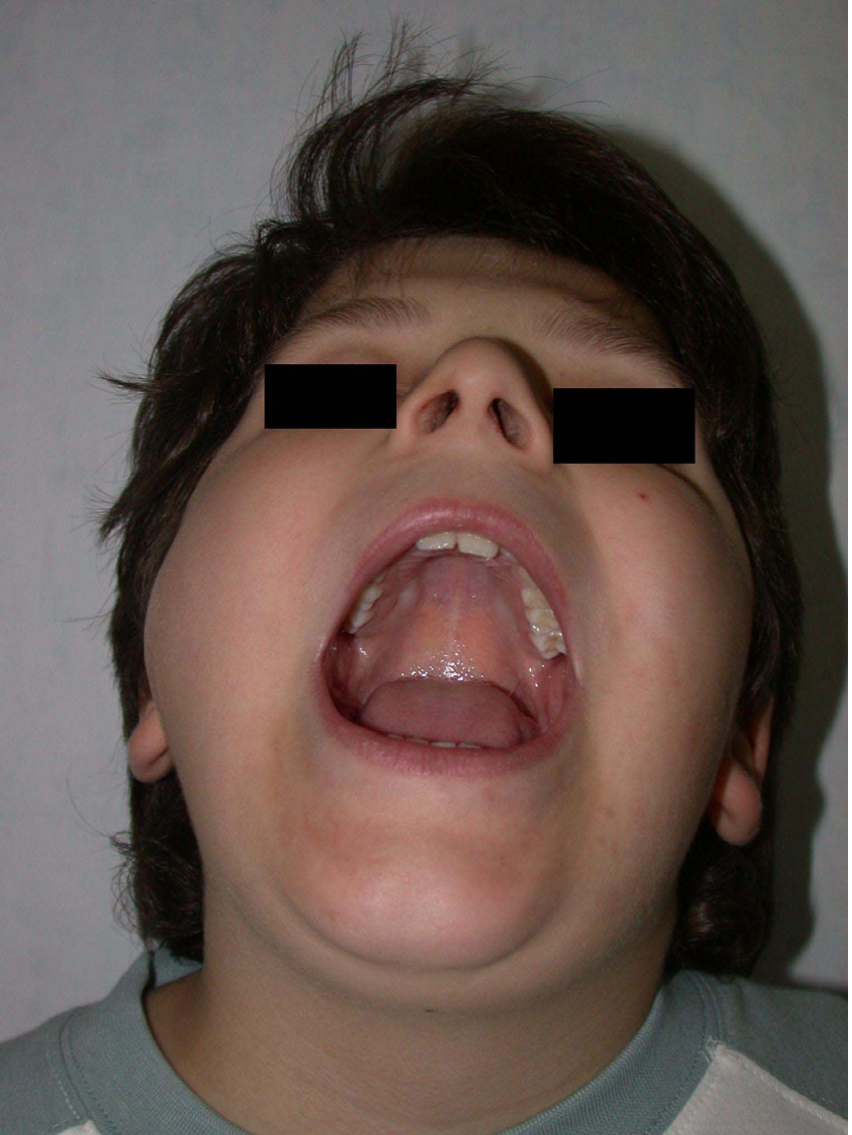
**Pre-operative image showing the severe swelling which depicted soft tissue invasion of the vascular Aneurismal Bone Cyst**. The patient was able to open widely.

A 10-year-old male patient presented with a painless mass in the right temporomandibular region. The swelling was evident since one month and it was hard and sensitive to palpation. The patient was able to open widely; the mandible could move in all axes without any limitation. The lesion caused facial asymmetry. There was no paresthesia in the area innervated by the right mandibular nerve or by the right facial nerve. The boy did not have any previous trauma in the swollen region [Figure 1].

**Figure 2:**
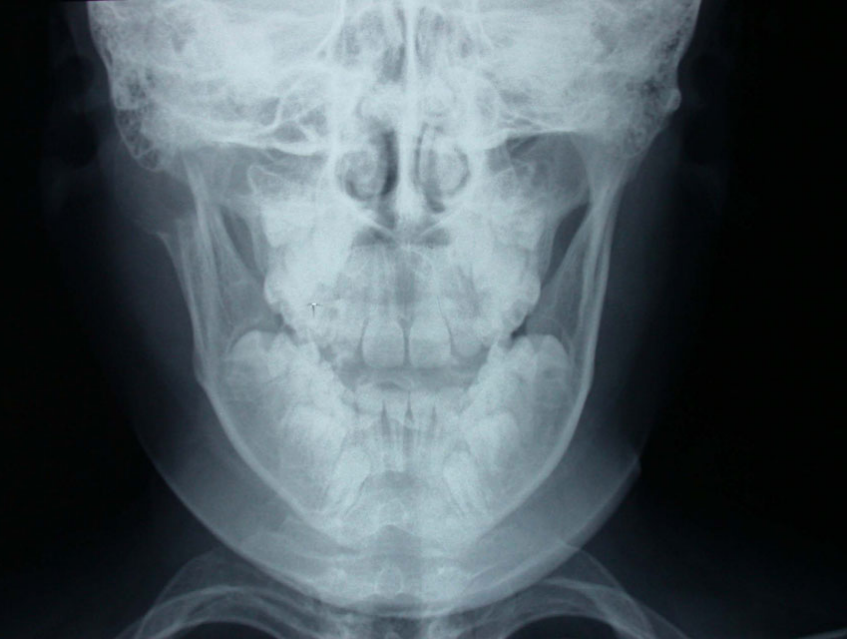
**Posterior-anterior radiography of the skull**. Radiolucent lesion of the right condyle.

**Figure 3:**
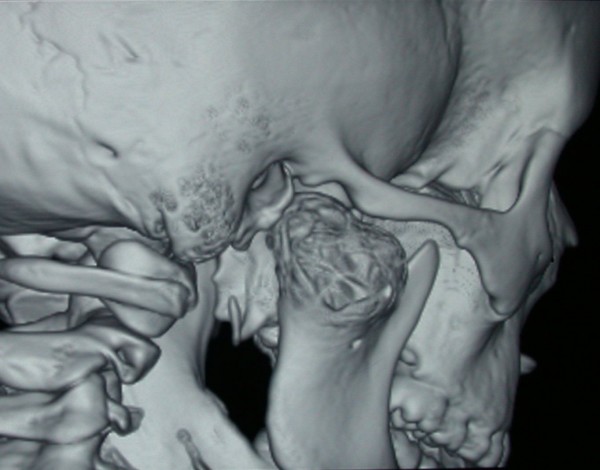
**Three-dimensional CT reconstruction showing the presence of a radiolucent and multilocular lesion in the condyle region, the coronoid and part of the ramus**. Note the honeycomb appearance and septae within the bony lesion.

**Figure 4:**
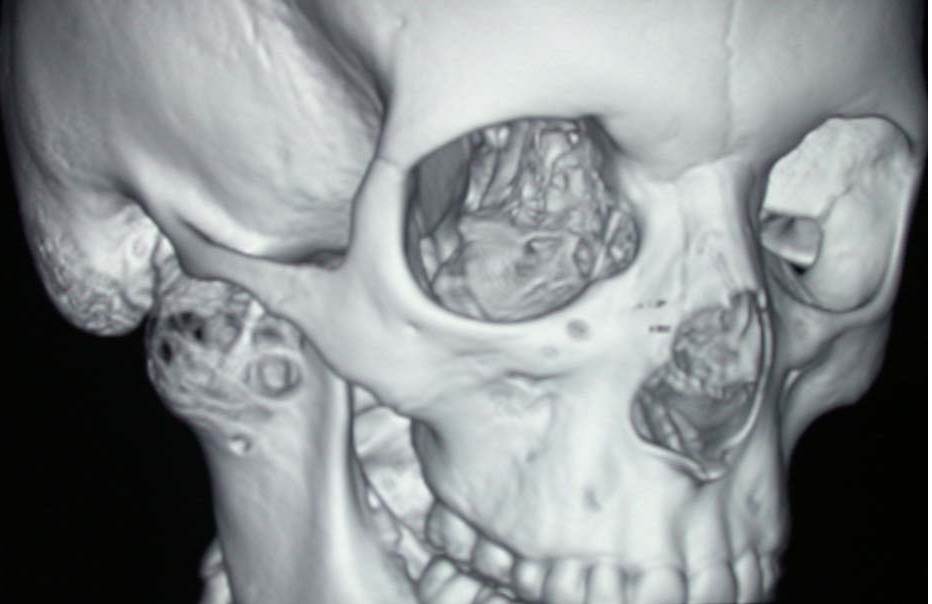
**Three-dimensional CT reconstruction showing the presence of a radiolucent and multilocular lesion in the condyle region, the coronoid and part of the ramus**. Note the honeycomb appearance and septae within the bony lesion.

**Figure 5:**
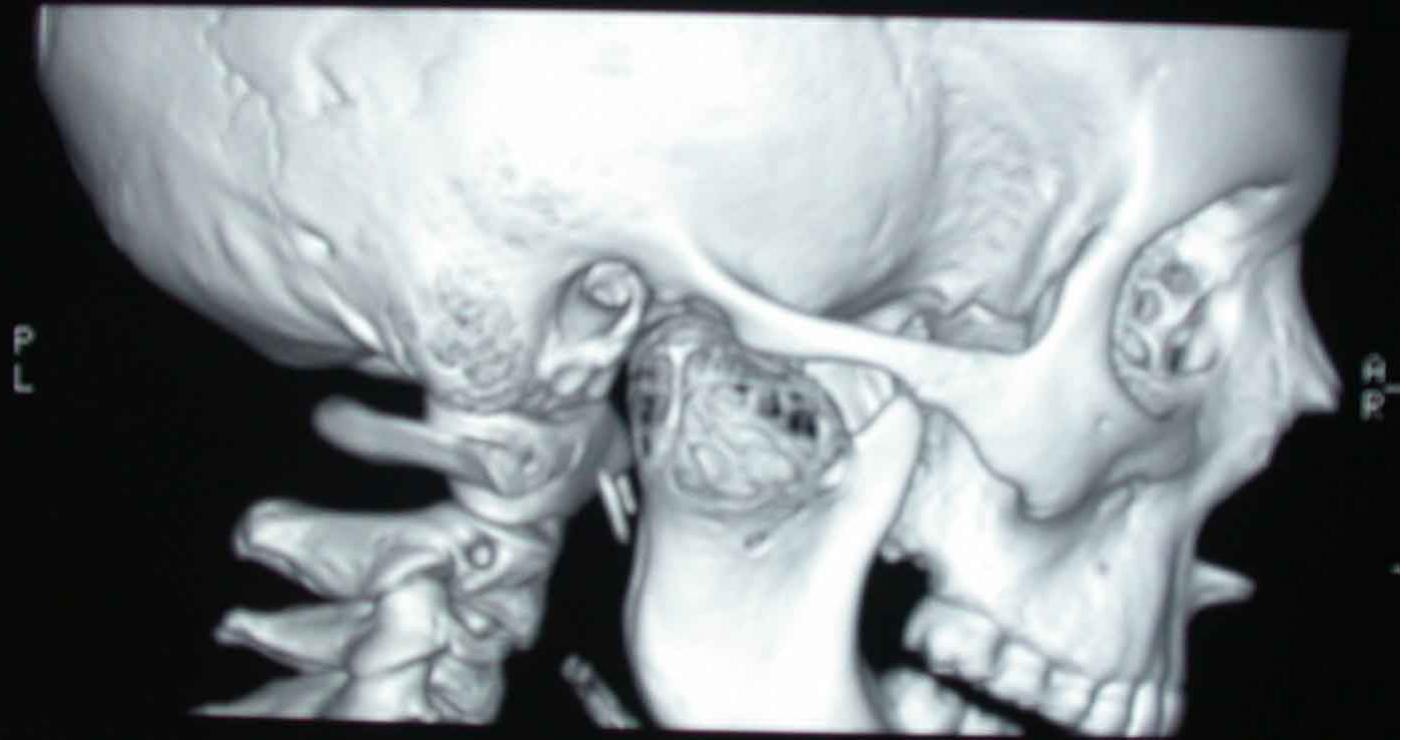
**Three-dimensional CT reconstruction showing the presence of a radiolucent and multilocular lesion in the condyle region, the coronoid and part of the ramus**. Note the honeycomb appearance and septae within the bony lesion.

**Figure 6:**
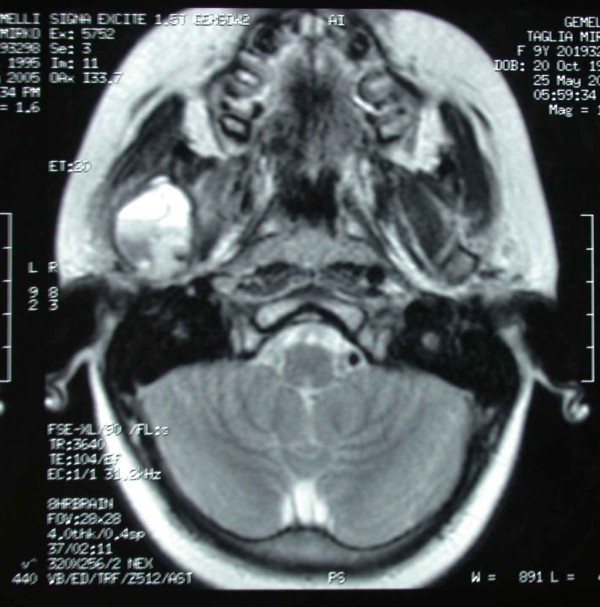
**Magnetic resonant imaging (MRI) showing a high-signal intensity within the lesion itself, with a low-signal intensity at the periphery**.

**Figure 7:**
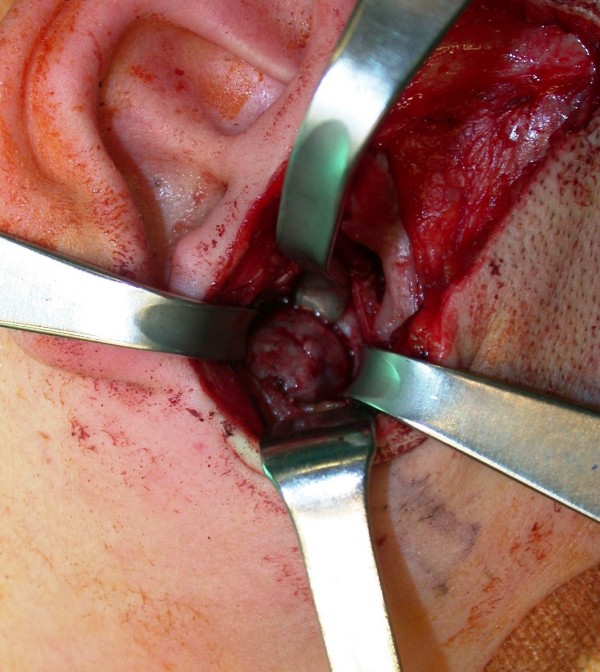
**Intraoperative exposure of the thin, translucent lateral cortical plate overlying the mandibular cyst**. Afterwards a low condylectomy, a coronoid process and mandibular ramus curettage were performed.

**Figure 8:**
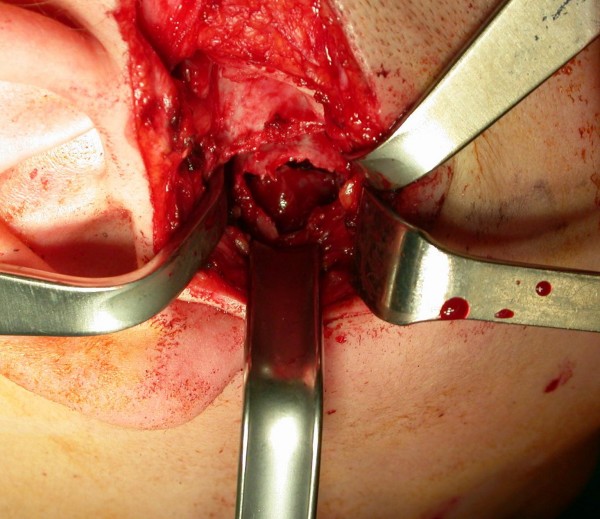
**Intraoperative exposure of the thin, translucent lateral cortical plate overlying the mandibular cyst**. Afterwards a low condylectomy, a coronoid process and mandibular ramus curettage were performed.

A posterior-anterior radiography of the skull was obtained revealing a radiolucent region in the condyle of the right mandible [Figure 2]. A three-dimensional CT reconstruction showed the presence of a radiolucent and multilocular lesion in the condyle region. It was possible to appreciate that the lesion had totally replaced the mandibular right condyle and part of the mandibular ascending ramus. The lesion substituted the bone marrow of these anatomical structures and the cortical bone was substantially expanded and perforated. The condyle was so ballooned that it was evident through extra oral examination. Part of the coronoid process was affected by the infiltrative osteolytic process [Figures 3, 4 and 5]. The magnetic resonant imaging (MRI) demonstrated a high-signal intensity within the lesion itself and a low-signal at the periphery [Figure 6]. The clinical presentation and the radiographic appearance of this lesion could have been associated to osteosarcoma, ameloblastma, myxoma or central giant cell granulomas therefore the decision for surgical excision was taken. The operation was performed under general anaesthesia. A preauricular incision was performed to identify the ATM joint capsule and thereafter the condyle. This structure appeared deformed and increased. The mandibular cortex overlying the cyst was noted to be thin and actually translucent in some places. Upon removal of the outer cortex of bone, thick greenish fluid was encountered, immediately followed by brisk hemorrhage which was difficult to control. Afterwards a low condylectomy was performed. The lesion underwent a complete surgical excision [Figures 7, 8]. The final histological diagnosis was aneurysmal bone cyst described as an expanding osteolytic lesion containing blood-filled spaces of variable size, separated by connective tissue constituted by bone trabeculae or osteoid tissue and many osteoclastic giant cells.

**Figure 9:**
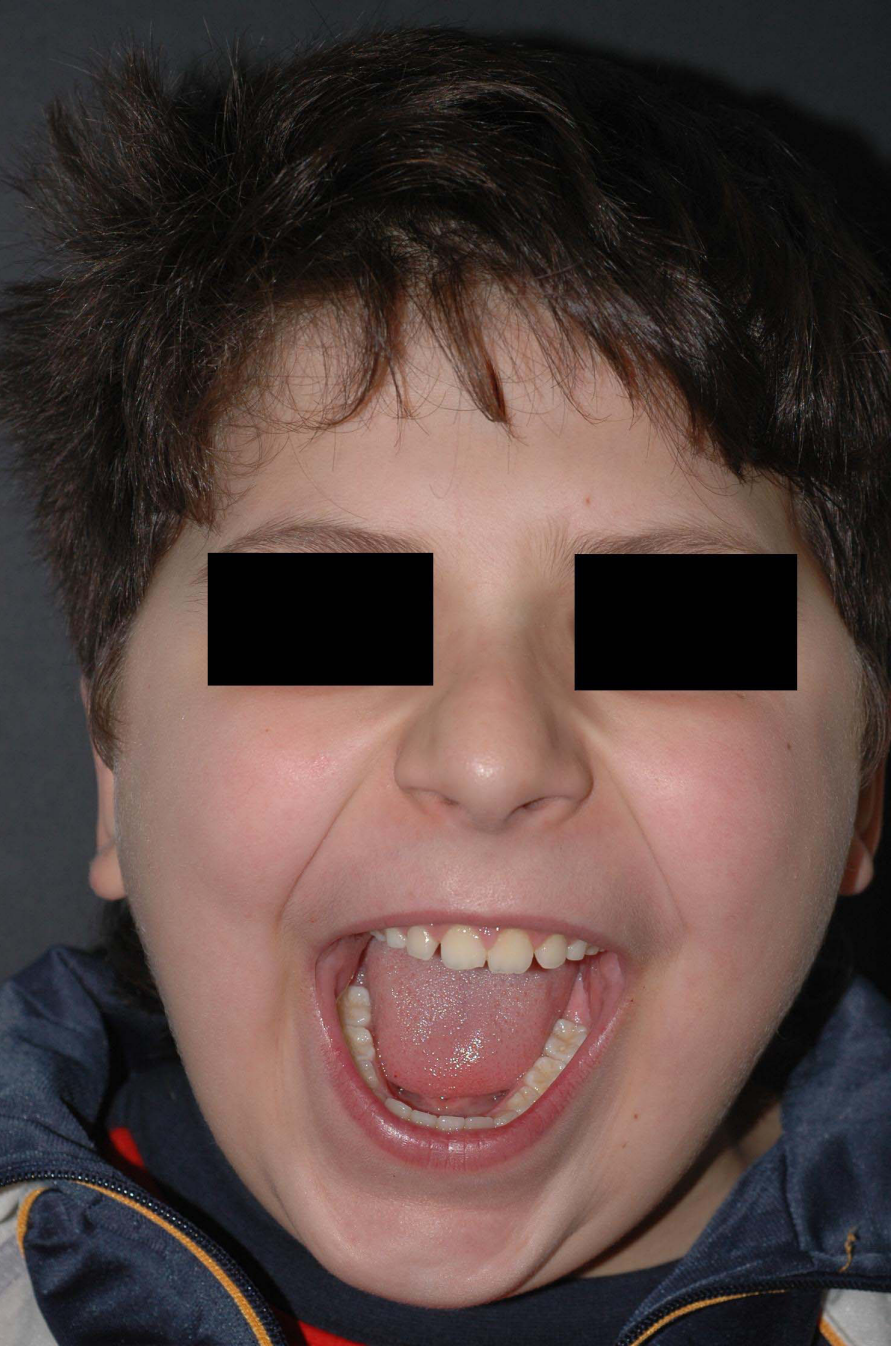
**Post-operative image showing the shifting of the mandible during opening towards the operated side**.

Patient's occlusion did not change after surgery however it was evident that the mandible shifted on the right side during opening [Figure 9]. The patient underwent throw physiotherapy and functional treatments with Bionator. This device helped the boy to have a good occlusion during his growth. The patient came monthly to our orthodontic department for functional exercises and to upgrade the Bionator.

## Discussion

Although the aneurysmal bone cyst is a lesion relatively common in the skeletal structure, is not usual to find it in the facial region of the skeleton. In 1893, Van Arsdale [15] called this lesion "homerus ossifying haematoma". In 1940 Ewing used the term "aneurismal" to describe such lesion [16]. In 1942, Jaffé and Lichtenstein [13] used the term "aneurismal cyst" and in 1950 they coined the term "aneurismal bone cyst" for the first time in literature. There are presented about 160 cases of aneurysmal bone cyst in the maxillofacial region in world literature [[1,3] and [4]].

Clinical presentations of the ABC range from mild, slowly expanding, semisolid growth causing slight facial asymmetry to a rapidly expanding vascular swelling causing extensive bone destruction and mimicking malignant lesions [10]. This lesion does not have a clear clinical specificity. The radiographic appearance of the vascular ABC typically demonstrates an expansive lesion with thinning of cortical plate and a honeycomb or soap bubble appearance [17,18].

These characteristics like the sudden growth, cortical destruction, osteoid formation, and tumour-like appearance, can easily cause confusion with malignancy.

It is important to differentiate the ABC from other pathologies that occur in the maxillofacial region. These include peripheral and central giant cell reparative granuloma, traumatic bone cyst, brown tumor of hyperparathyroidism, myxoma, fibrous dysplasia, desmoplastic fibroma, fibrous histiocytoma, hemangioma, osteogenic sarcoma, globulomaxillary cyst, hamangioendothelioma, and hemangiopericytoma [2,10]. The initial diagnosis can be made radiographically, with MRI being considered as the first choice diagnostic too. However definitive diagnosis requires histopathological examination of the surgical specimen.

It is important to note that, to make the diagnosis of aneurysmal bone cyst, it is mandatory to take into account patient's history, physical examination, radiographic and histopathologic evaluation [19].

The growth of the upper jaw, after surgery, was regular and seemed to not be influenced by the shifting movement of the mandible during opening. The forward translation of the healthy left condyle during opening operated by the left lateral pterygoid muscle was responsible for a mandibular shifting to the side were it was performed the condylectomy.

At present the patient is under control for any recurrence of the lesion. The differential diagnosis of aneurysmal bone cyst from malignant tumours is the main practical aspect of this osteolytic lesion.

Moreover the functional device Bionator is expected to stimulate the operated right side of the mandible for an additional growing. There is a vast documentation how functional appliances are used for hemifacial microsomia with excellent results [20]. The condyle and the ramus of the mandible have great potential of growing if opportunely stimulated [21].

A reconstruction of the right condyle will be taken in consideration as last option by the end of patient's growth only if the functional therapy will fail.

The authors warn that massive or excessive hemorrhage may complicate surgery and be difficult to control [5,10]. Similar intra-surgery complication have been described in others publications. Good surgical exposure and control of any feeding vessel that can be identified close to the lesion facilitate excision, minimize blood loss, and aid in the preservation of vital structures. This case demonstrated the aggressive and destructive behaviour common to vascular ABC of the jaw.

The treatment of this lesion consist of complete surgical excision, it demonstrates a low recurrence rate. It has been proposed radiation therapy however the risk of subsequent malignant degeneration is present. It has been reported sarcoma arisen within radiated ABC [[1,3] and [4]]. Even curettage of the cysts has a recurrence rate as high as 50%. Technical difficulties in entirely removing very large lesions can be the explanation for very different recurrence rate within literature [1-23]. This article suggests how a complete surgical resection can definitively eradicate this aggressive bone lesion with a quite high recurrence rate. In conclusion surgical excision is the treatment of choice and the differential diagnosis of ABC from malignant tumours is the most important clinical aspect. No recurrence was observed at a 3 years follow-up.

## Consent

Written informed consent was obtained from the patient and patient's parents for publication of this case report and accompanying images. A copy of the written consent is available for review by the Editor-in-Chief of this Journal.

## Competing interests

The authors declare that they have no competing interests.

## Authors' contributions

SP, GG and PFA performed the surgery and carried out the case study. PFA and RB wrote the article. AM reviewed scientific literature for this mandibular cyst. All the authors have read and approved the manuscript.
